# Field-grown transgenic wheat expressing the sunflower gene *HaHB4* significantly outyields the wild type

**DOI:** 10.1093/jxb/erz037

**Published:** 2019-02-06

**Authors:** Fernanda Gabriela González, Matías Capella, Karina Fabiana Ribichich, Facundo Curín, Jorge Ignacio Giacomelli, Francisco Ayala, Gerónimo Watson, María Elena Otegui, Raquel Lía Chan

**Affiliations:** 1 Estación Experimental Pergamino, Instituto Nacional de Tecnología Agropecuaria (INTA), Pergamino, Buenos Aires, Argentina; 2 CITNOBA, CONICET-UNNOBA, Pergamino, Buenos Aires, Argentina; 3 Instituto de Agrobiotecnología del Litoral, Universidad Nacional del Litoral – CONICET, Facultad de Bioquímica y Ciencias Biológicas, Santa Fe, Argentina; 4 INDEAR/BIOCERES, Rosario, Argentina; 5 CONICET-INTA-FAUBA, Estación Experimental Pergamino, Facultad de Agronomía Universidad de Buenos Aires, Buenos Aires, Argentina

**Keywords:** Drought tolerance, grain yield determination, HaHB4, sunflower transcription factor, transgenic wheat, water use efficiency, wheat field trials

## Abstract

HaHB4 is a sunflower transcription factor belonging to the homeodomain-leucine zipper I family whose ectopic expression in Arabidopsis triggers drought tolerance. The use of PCR to clone the *HaHB4* coding sequence for wheat transformation caused unprogrammed mutations producing subtle differences in its activation ability in yeast. Transgenic wheat plants carrying a mutated version of *HaHB4* were tested in 37 field experiments. A selected transgenic line yielded 6% more (*P*<0.001) and had 9.4% larger water use efficiency (*P*<0.02) than its control across the evaluated environments. Differences in grain yield between cultivars were explained by the 8% improvement in grain number per square meter (*P*<0.0001), and were more pronounced in stress (16% benefit) than in non-stress conditions (3% benefit), reaching a maximum of 97% in one of the driest environments. Increased grain number per square meter of transgenic plants was accompanied by positive trends in spikelet numbers per spike, tillers per plant, and fertile florets per plant. The gene transcripts associated with abiotic stress showed that HaHB4’s action was not dependent on the response triggered either by RD19 or by DREB1a, traditional candidates related to water deficit responses. HaHB4 enabled wheat to show some of the benefits of a species highly adapted to water scarcity, especially in marginal regions characterized by frequent droughts.

## Teaser

Transgenic wheat expressing the sunflower transcription factor HaHB4 outyields the wild type by 6% when tested in the field during 9 years across 37 contrasting environments.

## Introduction

Plants have evolved molecular mechanisms to deal with stress conditions, enabling their survival and reproduction. Among abiotic stress factors, drought is the major limiting constraint on agricultural productivity (Wang *et al.*, 2003). Drought tolerance has been used as a key parameter to select transgenic stress-tolerant model plants and crops (Araus and Cairns, 2014). However, drought tolerance and yield do not always follow the same trend. Most research on drought effects has used Arabidopsis plants grown in controlled growth conditions and evaluated mechanisms relating to survival rather than to stress tolerance (Zhang *et al.*, 2004). In a review of more than 1000 scientific papers on water deficit at the molecular level, Passioura (2012) detected that 40% used Arabidopsis and the enhanced survival of transformed plants was simply explained by their reduced size and concomitant slower water uptake compared with the wild type (WT) (Morran *et al.*, 2011). Similarly, improved drought stress tolerance that involved stomatal closure caused improved water use efficiency but at the expense of decreased photosynthetic rate and biomass production. Consequently, the reported success in severe stress tolerance did not result in yield improvement but frequently in yield decrease in mild stress or standard growth conditions.

In the same way, performing a deep phenotypic analysis of 25 independent Arabidopsis transgenic events previously described as drought-tolerant, it was reported that none of them showed significant genotype-specific responses with moderate drought stress (Skirycz *et al.*, 2011). Authors of such research concluded that enhanced survival under severe drought is not a good indicator of improved growth performance or yield under mild stress conditions, the most frequent situation (Skirycz *et al.*, 2011). On the other hand, a few transgenic events have been described as conferring stress tolerance without yield penalties. This is the case for HVA1, a late-embryogenesis-abundant protein introduced as a transgene in rice, directed by a synthetic abscisic acid (ABA)/stress-inducible promoter; these rice plants exhibited modified root architecture under abiotic conditions (Chen *et al.*, 2015). Another research group introduced a b-Zip transcription factor in potato plants, which showed improved yield compared with controls (Moon *et al.*, 2015). Another example is represented by peanut plants expressing *DREB1A*, which showed a lower yield penalty than controls in field trials (Bhatnagar-Mathur *et al.*, 2014). Notably, in all these cases the species barrier was crossed. When the wheat genes *TaDREB2* and *TaDREB3* encoding transcription factors were expressed in wheat and barley, under the control of either constitutive or inducible promoters, the transgenic plants showed improved survival under severe drought conditions relative to non-transgenic controls. However, these plants exhibited also slower growth, delayed flowering and low grain yields (Morran *et al.*, 2011). Drought-tolerant crops with a demonstrated yield increase in field trials are almost absent from the scientific literature, and only a few transgenic technologies applied to other species have up to now given a commercial product. This is the case for maize MON87460×NK603 expressing the bacterial RNA chaperones CspB and CspA, which conferred cold, heat, and drought tolerance to rice plants too (Castiglioni *et al.*, 2008; http://www.isaaa.org/gmapprovaldatabase/). Moreover, other transgenic events yielding abiotic stress tolerance have been given approval with regard to food safety and biosafety, but to the best of our knowledge these products have not been released to the market yet. In this last group is the sugarcane NXI-4T expressing a betaine gene that acts as an osmoprotectant and confers the ability to produce more sucrose than controls under water deficit stress conditions (Sugiharto, 2017).

Among the severe stress-tolerant events tested in model plants, a significant percentage are represented by transcription factors (TFs). Plant TFs, divided into several families and subfamilies according to structural and functional features, play important roles regulating whole stress response cascades and are considered key targets for improving stress tolerance in crop plant species (Century *et al.*, 2008).

The homeodomain-leucine zipper I (HD-Zip I) family of TFs is unique to plants, its members have been characterized as active players in the adaptive response to several abiotic stresses, and the expression of several of them is regulated by drought, salt, and ABA in different tissues/organs (Henriksson *et al.*, 2005; Ariel *et al.*, 2007). Although most studies were performed with proteins from model plants, in particular from Arabidopsis (Perotti *et al.*, 2017), HD-Zip I proteins from other species have been characterized too. Such is the case of *Medicago truncatula* MtHB1 (Ariel *et al.*, 2010), sunflower HaHB1 (Cabello *et al.*, 2012; Cabello and Chan, 2012), and maize *Zmhdz10* (Zhao *et al.*, 2014), which when expressed in a different species conferred enhanced tolerance to abiotic stresses. It is important to note that, for example, *HaHB1* transgenic Arabidopsis exhibited fewer penalties than control plants under drought stress conditions because the molecular mechanism triggered by this TF did not involve stomatal closure but cell membrane stabilization (Cabello and Chan, 2012). In the same way and crossing the interspecific barrier, it has been found that the ectopic expression of *AtHB16* in bahiagrass and related species suppressed or reduced the formation of seed-heads leading to an increase in the number of vegetative tillers per plant and improved tolerance to abiotic stresses (Altpeter and Zhang, 2010). Proteins belonging to the HD-Zip I family show high conservation of the HD-Zip domain and also conserved motifs outside this domain. Such features allowed them to be resolved into six phylogenetic groups (Arce *et al.*, 2011).

Notably, species of the Asteraceae show divergent members that could not be resolved into any of these six clades. The sunflower HaHB4 is one of these divergent TFs in this family exhibiting an atypical short carboxy terminus (Arce *et al.*, 2011). The expression of *HaHB4* is induced by ABA, water deficit, ethylene, and jasmonic acid, among other environmental and hormonal factors (Gago *et al.*, 2002; Dezar *et al.*, 2005; Manavella *et al.*, 2006; Manavella *et al.*, 2008*a*, *b*). The ectopic expression and overexpression of this gene in Arabidopsis, driven by its own promoter or by other inducible or constitutive ones, resulted in plants tolerant to drought, salinity, and herbivory (Dezar *et al.*, 2005, Manavella *et al.*, 2008*a*, *b*) and caused an increase in xylem area and disruption of the vein symmetry of the leaves (Moreno Piovano *et al.*, 2017). Drought tolerance is linked to the inhibition of ethylene perception and does not involve ABA-mediated stomatal closure. Moreover, stomatal closure in *HaHB4* Arabidopsis transgenic plants was delayed with respect to WT controls (Manavella *et al.*, 2006). Two wheat HD-Zip I members, TaHDZipI-2 and TaHDZipI-5, were characterized and introduced as transgenes in wheat and barley under constitutive promoters. These transgenic plants showed improved tolerance to drought and frost stresses; however they exhibited undesirable phenotypic characteristics such as reduced size, biomass, and yield (Kovalchuk *et al.*, 2016; Yang *et al.*, 2018). Based on the described observations for HaHB4 Arabidopsis transgenic plants, we hypothesized that the molecular mechanisms displayed by this transgene could be conserved in crops.

Wheat (*Triticum aestivum L.*) is an autogamous crop belonging to the Poaceae and used mostly to produce human food (ca. 74%, whereas 16% is destined for animal feed and the rest for industrial applications; Willenborg and Van Acker, 2010). As it provides 20% of the calories of the human diet, its production (>700 million tons) is considered vital to ensuring global food security (Chand, 2009; Tweeten and Thompson, 2010). The rise in world population, expected to reach ca. 10 billion people by 2050 (https://population.un.org/wpp/Graphs/Probabilistic/POP/TOT/), together with the improvement in diet quality, will increase wheat demand in the near future (Borlaug, 2007; Chand, 2009). To fulfill this demand, it has been calculated that global wheat production should double in the next decades (Hall and Richards, 2013). This goal, however, is difficult to achieve with current breeding approaches (Hall and Richards, 2013) and considering the expected negative effects of climate change (IPCC, 2014). With regard to breeding, the second generation of transgenic crops is projected to mitigate abiotic stress effects, but most evaluated events have failed to translate benefits observed in controlled environments to field conditions (Passioura, 2012). With regard to climate, wheat yield gaps (i.e. the difference between potential and actual yield) vary extensively depending on the production area (www.yieldgap.org), with water deficits of variable duration and intensity as one of the main determinants of yield loss. Increasing crop tolerance to abiotic stress is an avenue to reduce these gaps and give increased crop production. Despite the importance of wheat as a food and feed staple, significant investments in crop improvement have been unattractive for technology developers. As a self-pollinated crop, wheat has historically faced difficulties in royalty collections due to farmer-saved seeds. GM events have significantly improved economics for developers in other self-pollinated crops such as soybean, but anti-GM consumer groups have boycotted any similar attempts in wheat, to the point where it was recently described as ‘the cereal abandoned by GM’ (Wulff and Dhugga, 2018).

In this work we describe obtaining and testing of transgenic wheat expressing the sunflower transcription factor HaHB4, which experienced a series of point mutations during cloning. In yeast, the mutant version of HaHB4 presented transactivation activity and the ability to dimerize with other HD-Zip I members. The performance of transgenic wheat plants was analysed at different scales in 37 field experiments that were organized in four groups depending upon the scope of the study. The first group (six experiments out of 37) was used for the selection of the best performing transgenic line with respect to the WT parental cultivar. Grain yield and its two main components (grain number per m^2^ and individual grain weight) were evaluated in this group. The second group was used for the comparison between the best performing transgenic line and the WT parent in the testing net developed by INDEAR (www.indear.com.ar). It included all 37 experiments across a wide range of environments in Argentina. Grain yield and anthesis date, as well as grain yield components (spikes per m^2^, grain numbers per m^2^, and grain weight) were assessed in this second group. The third group (four experiments out of 37) was used for a more detailed analysis of the ecophysiological determinants of grain yield (i.e. biomass production and partitioning). Finally, in the fourth group (one experiment out of 37), floral morphology and detailed crop phenology were evaluated, together with expression levels of the transgene and genes related to abiotic stress responses. Expression levels were correlated with grain yield determinants. Argentina had two advantages for the proposed research: (i) a monsoonal rainfall distribution (i.e. larger in the warm than in the cool period of the year), which usually exposes wheat crops to a variable degree of water deficit depending on the region, and (ii) a non-hostile position towards transgenic technologies, which facilitates on-farm experimentation.

## Materials and methods

### Genetic constructs

*pIND-HB4*: this construct was obtained by cloning *HaHB4* in a plasmid (derived from pUC8) bearing a 1992 pb *Pst*I fragment containing the promoter (899 bp), the 5′-untranslated region (83 bp), and the first intron (1010 bp) of maize ubiquitin followed by the coding sequence (CDS) of *HaHB4.2* and the nopaline synthase terminator of *Agrobacterium tumefaciens*, which includes the polyadenylation signal. The native *HaHB4* cDNA cloned at the *Bam*HI/*Sac*I sites of *pBluescript SK−* (Gago *et al.*, 2002) was used as the template in a PCR reaction with oligonucleotides H4-F and Transf2 (see Supplementary Table S1 at *JXB* online), which included initiation and stop codons. The construct was checked by sequencing (Macrogen, Korea) and accidental mutations detected. Since this construct has already been introduced in plants, and in order to test the putative effect of such mutations, other modified versions of HaHB4 cDNA were obtained by successive PCR reactions with oligonucleotides H4m-F and H4m-R, Transf1, H4m-R1, H4m-F1, and Tranf2 (Supplementary Table S1). The PCR products were cloned in a *pGEM T-easy* vector (Promega) having flanking sites *Bam*HI and *Sac*I, used to replace the native HaHB4 cDNA. The resulting amino acid sequences are described in the Results section.

*pIND4-Bar*: this plasmid was constructed in a similar way to *pIND4-HB4* but instead of the CDS of HaHB4, the bar gene of *S. hygroscopicus* was cloned at the *Bam*HI site.

The constructs used for Y2H, namely AtHB1, AtHB7, AtHB12, and AtHB13 lacking the CTR as a Gal4-DNA-binding domain fusions, were previously described in Capella *et al.* (2014).

### One- and two-hybrid assays in yeast

Transactivation capability was evaluated by measuring β-galactosidase using *o*-nitrophenyl-β-galactoside as substrate.

HaHB4.2 cDNA was cloned into the *pGADT7* vector and fused to the activation domain of the yeast transcription factor GAL4. The resulting expression construct was then transformed into *Saccharomyces cerevisiae* strain AH109, and a two-hybrid assay was performed using the yeast expressing the modified protein and four Arabidopsis HD-Zip proteins with deleted carboxy termini that were previously cloned in the *pGBKT7* vector and fused with the GAL4 binding domain therein.

### Wheat transformation and transgenic line selection

Wheat transformation was carried out by INDEAR S.A. (Rosario, Argentina) in 2005. Essentially, the transformation method used was that developed by Pastori *et al.* (2001). Sterile embryos of wheat cv. Cadenza (WT) were bombarded using a PDS-1000/He (Biolistic® PDS-1000/He particle delivery system, Bio-Rad) device and gold-coated microparticles bearing two different plasmids: *pIND4-HB4* and *pIND4-Bar*. The first one contained the modified version 2 of HaHB4 (called HaHB4.2) and the second contained the bar gene of *Streptomyces hygroscopicus* (Thompson *et al.*, 1987) encoding phosphinothricin *N*-acetyl transferase, which confers ammonium glufosinate tolerance. The selective marker was ammonium glufosinate, and 3 weeks after the bombardment, green seedlings were put on soil until harvest.

A total of 12 transgenic (TG) events were obtained. From each event, 16–20 individuals were grown in pots in a greenhouse (T0). The presence of *HaHB4* was checked by PCR, and five events were selected based on a χ^*2*^ test (*P*≥0.15). The first multiplication (T1 seed) was carried out in 2007. A total of 60 lines derived from selfing of the selected events were sown under a hail shelter and tested for segregation (3:1 segregation in T1). During the vegetative stage, plants were sampled for PCR analysis to identify homozygous lines. Lack of negative segregants among the sampled progeny (at least five individuals sampled per line) was used as an indicator of homozygosis. Five homozygous lines (T2) were selected and evaluated for growth (e.g. plant height) and development (i.e. phenology by the Zadoks scale; Zadoks *et al.*, 1974) characterization during 2008. At least 10 plants of each event were included in the analysis (Supplementary Table S2). Enough seed of three of these lines was obtained for grain yield evaluation (T3) in the first group of field experiments performed during 2009 and 2011.

### Experimental layout and crop husbandry of field trials

A total of 37 field experiments were carried out (Supplementary Table S3), derived from the combination of 9 years (2009–2017) and 13 sites covering a wide range of latitudes (27°14′S to 39°52′S) and longitudes (57°40′W to 63°30′W), with replicated experiments in some cases (e.g. two different sowing dates in Monte Buey and Carmen de Areco 2012, and two independent experiments in Villalonga 2009 and 2010). Actual environmental conditions experienced by crops in each experiment are summarized in Supplementary Table S3, including the reproductive phase that encompasses the critical period for grain number determination (i.e. the period between anthesis−20 d to anthesis+10 d) and the grain-filling period (from anthesis to maturity). Delayed sowing dates (i.e. later than 10 June for the long-cycle WT Cadenza) were used in several experiments to increase the probability of water deficit and/or heat stress during the reproductive phase. Based on the National Wheat Net Trials (www.inase.gov.ar), this goal was largely met because mean heading date took place ca. 19 d later than the expected heading date of long-cycle wheat cultivars (data not shown).

Experiments were organized in four groups depending upon the scope of the study. The first group (Group 1) included six of the 37 experiments, and aimed at the selection of the best performing transgenic line with respect to the WT parental cultivar. The three events mentioned before together with the WT were evaluated in this group. The six experiments corresponded to (i) the Villalonga site (Supplementary Table S3) in 2009 (irrigated and non-irrigated) and 2011, (ii) the Monte Buey site in 2009, and (iii) the Daireaux and Villa Saboya sites in 2011. Results from Group 1 distinguished the transgenic line identified as Ta.IV.ii.a.12 as the best performing (*P*<0.05), which was registered as IND-ØØ412-7. The second group of experiments (Group 2) aimed at the comparison between the best performing transgenic line (IND-ØØ412-7) and the WT Cadenza, and included all 37 experiments. The third group (Group 3) included four of the 37 experiments, three during 2016 (Monte Buey, Pergamino and Roldán) and one during 2017 (Pergamino). This group aimed at a detailed analysis of the ecophysiological determinants of grain yield (described next). The last group (Group 4) corresponded to only one of the 37 experiments (Pergamino in 2017), where floral morphology and detailed crop phenology were evaluated together with expression levels of the transgene and genes related to abiotic stress responses (described next).

In each of the 37 experiments, cultivars were machine-sown in a randomized complete block design with three replicates. Each unit (plot) had seven rows of 5 m length with a distance of 0.2 m between rows (i.e. 7 m^2^ per plot). The exceptions were experiments developed at Villalonga (2009 and 2011), where plots were five rows of 5 m length, and at Pergamino in 2017, where plots were 7 m length (i.e. 9.8 m^2^ per plot).

Crops in all experiments were kept free of weeds, insects, and diseases by means of recommended chemical controls.

### Grain yield, water use efficiency, biomass production, and grain yield components

Whole plot grain yield (GY_P_) was obtained by (i) machine-harvest of the five central rows of each plot (i.e. 3 m^2^) in all experiments performed between 2009 and 2016, or (ii) hand-harvest of 1.5 m (i.e. 0.3 m^2^) in 2017. For Group 2, water use efficiency (WUE) was calculated as the per plot grain yield adjusted for the total rainfall received during the crop cycle, expressed in kg ha^−1^ mm^−1^ (WUE_GY,R_=GY/Rainfall; Sinclair *et al.*, 1984). Grain yield components (spikes m^−2^, grains m^−2^, and grain weight) were estimated in all plots, except spikes m^−2^ in experiments conducted in 2009 and 2010 (i.e. 28 out of 37 environments). The number of spikes present in 1 m (0.2 m^2^) was counted in the central row of each plot before harvest.

For experiments developed between 2009–2015, individual grain weight (GW) of each plot was estimated from a subsample of at least 400 grains, and grain number m^−2^ computed as the quotient between GY_P_ and GW.

The ecophysiological determinants of grain yield (Fischer 1983, 1985), namely total biomass production at maturity and harvest index (HI), were evaluated in experiments of Group 3. At maturity, an area of 0.2 m^2^ (2016) or 0.3 m^2^ (2017) was harvested in each plot for the estimation of HI, which was computed as the quotient between grain yield of the sampled area (GY_S_) and total biomass of the sampled area (*B*_S_). The number of grains present in each of these samples was counted (GN_S_) and GW was computed as the quotient between GY_S_ and GN_S_. Total biomass of the whole plot (*B*_P_) was estimated as the quotient between GY_P_ and HI.

Crop growth rate (CGR, in g m^−2^ d^−1^) during the 30 d period before anthesis was estimated only at Pergamino during 2016 and 2017, by sampling the total biomass in 0.2 m^2^ of each plot on ca. 30 d before anthesis and at anthesis.

### Phenology and floral development evaluation

At Pergamino in 2017 (Group 4), phenology was surveyed every 3 d for the correct estimation of the stages of beginning of stem elongation (first node detectable to the touch at ca. 1 cm above the tillering node), booting, flag leaf expanded, heading, and anthesis (Zadoks *et al.*, 1974). Twenty consecutive plants were evaluated along the central row of each plot to establish initiation of stem elongation. For determining other growth stages, a 2 m stretch of the central row of each plot was visually inspected. Plots were considered in a particular growth stage when at least 50% of inspected plants reached that stage.

For the study of apex development three plants per plot were removed twice weekly from beginning of tillering until beginning of stem elongation. The plants were dissected under a binocular microscope and the apex stage was surveyed following Kirby and Appleyard (1987). At anthesis, the floret stage of each floret primordium along the spike was assessed following the scale of Waddington *et al.* (1983). The florets were counted as fertile when they were in stage W>9.25 and anthers were green (González *et al.*, 2003). For this purpose, three representative plants from each plot were sampled and main stems and tiller spikes were counted and separated from the rest of the plant. Using a binocular microscope, fertile florets within each spikelet were counted to obtain the number of spikelets spike^−1^ and fertile florets spike^−1^, in the main shoots and tillers. Afterward the spikes were dried in an air-forced oven to estimate spike weight at anthesis.

### RNA isolation and expression analyses by real time RT-PCR

RNA for RT-qPCR was extracted from each plot of the experiment performed at Pergamino during 2017 (Group 4), i.e. three biological replicates per genotype including tissue from eight different plants each. TRIzol® reagent (Invitrogen) was used for extraction, according to the manufacturer’s instructions. RT-PCR analyses were conducted essentially as described previously (Cabello *et al.*, 2012). RNA levels were quantified by normalization with *ACTIN* and *UBIQUITIN* transcript levels according to the ΔΔ*C*_t_ method. Each biological replicate was tested in triplicate (i.e. three technical replicates) and used to calculate standard error. Differences were considered significant when the *P*<0.05 (Student’s *t*-test). Specific oligonucleotides for *HaHB4* and wheat *LOX2*, *RD19*, *DREB1a*, *ACO2*, and *AOS* were designed and are shown in Supplementary Table S1.

### Statistical analyses

Differences in grain yield and its components (grain number and grain weight) among (WT Cadenza and all TG events in Group 1) and between cultivars (WT Cadenza and IND-ØØ412-7 in Group 2) were analysed using nested analyses of variance (ANOVAs), with genotypes (G) and environments (E) as fixed factors and replicates nested within environments. Each experiment was considered an independent environment. Differences between means were analysed by means of the Tukey test. The same procedure was used for the evaluation of water use efficiency (Group 2), phenological events (Group 4), and grain yield, its physiological determinants and its components (Group 3). Means ± standard error were used for comparison of floral development in Group 4. The relationship between variables was tested by correlation and regression analyses.

### Accession numbers

For wheat genes, accession numbers are from GrainGenes database (The *T. aestivum* cv. Cadenza (Earlham Institute Scaffolds, 2017) wheat collection at https://wheat.pw.usda.gov/GG3/): *TaLOX2*: scfld336632_5BS, scfld361901_5DS; *TaRD19*: scfld374347_5DL, scfld346111_5BL, scfld321097_5AL; *TaDREB1a*: scfld322800_5AL; *TaACO2*: scfld432067_6BL; *TaACTIN*: scfld453873_6DL; scfld348405_5BL.

For sunflower *HaHB4*, accession numbers in EMBL, GenBank and DDBJ Nucleotide Sequence Databases are AF339748 and AF339749.

## Results

### Mutations in the sunflower *HaHB4* CDS revealed the importance of particular amino acids in transactivation ability

**Fig. 1.**
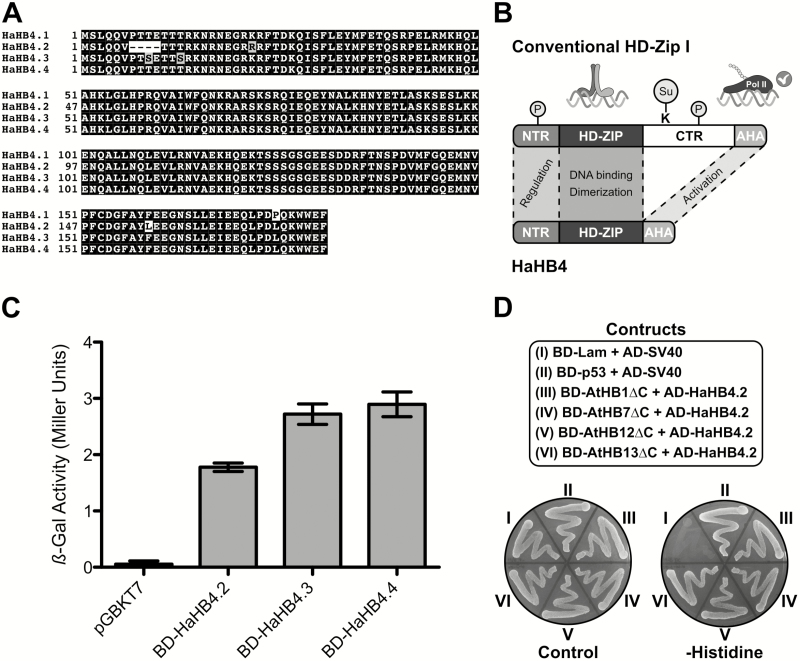
HaHB4.2 binds HD-Zip I transcription factors and activates transcription in yeast. (A) Multiple sequence alignment of the different HaHB4 isoforms. Darker background corresponds to enhanced conservation and numbers highlight the positions of amino acids. HaHB4.1 represents the WT form of sunflower HaHB4. (B) Schematic representation of the domains exhibited by most HD-Zip I and HaHB4. AHA, aromatic and large hydrophobic residues embedded in an acidic context; CTR, carboxy-terminal region; HD-Zip, homeodomain-leucine zipper; NTR, amino-terminal region; P, phosphorylation; Su, SUMOylation. (C) HaHB4 isoforms act as transcriptional activators in yeast-one hybrid assays. β-Galactosidase activity was quantified in Miller units and error bars represent standard deviation of three independent technical triplicates. (D) HaHB4.2 interacts with HD-Zip I from Arabidopsis. Yeast two-hybrid analysis of HaHB4.2 (as a Gal4-activating domain fusion; AD) with AtHB1, AtHB7, AtHB12, and AtHB13 lacking the CTR as a Gal4-DNA-binding domain (BD) fusion). Growth on plates without histidine indicates protein interaction. SV40 large-T antigen (AD-SV40) with either human lamin C (BD-Lam) or murine p53 (BD-p53) was used as negative and positive controls, respectively.

To test a putative beneficial effect of the sunflower TF HaHB4 in wheat, its cDNA was amplified by PCR and introduced in suitable genetic constructs. Sequencing of the genetic construct revealed several mutations that occurred during cloning. This new construct was named HaHB4.2. Since we already knew the importance of conserved motifs outside the HD-Zip domain (Arce *et al.*, 2011), additional mutations in similar locations were performed on HaHB4 and an alignment of four different HaHB4 modified clones was carried out (Fig. 1A). Four amino acids were lacking in the N-terminus of HaHB4.2 and a proline was replaced by a leucine in the C-terminus of HaHB4.2, -3 and -4. Moreover, HaHB4.3 exhibited two serines instead of two threonines in the N-terminus and HaHB4.2 presented two other conservative changes: an arginine instead of lysine in the N-terminus and a leucine instead of phenylalanine in the C-terminus (Fig. 1A). Since HaHB4 is a divergent member of the family and presents a very short C-terminus compared with other family members (Fig. 1B), it was difficult to predict the effect of such changes in the activity of this TF. It is noteworthy that the open reading frame was not altered with these mutations.

Even when the initial mutations were accidentally introduced, we decided to test both the ability to transactivate and the phenotypic effects of the obtained sequence variants in yeast and Arabidopsis, respectively. A simple yeast hybrid assay was carried out using constructs in which the different variants of HaHB4 were cloned in the pGBKT7 vector in operative association with the GAL4 DNA binding domain. The results indicated that the transactivation ability was affected in modified versions of HaHB4: HaHB4.4 and HaHB4.3 were more active than HaHB4.2 (Fig. 1C). Arabidopsis plants transformed with such different versions of HaHB4 were tested for drought tolerance; however, no significant differences were observed between the different genotypes; all of them behaved as previously reported for WT HaHB4 (not shown).

Similarly, in order to determine the ability of the modified HaHB4 protein to heterodimerize, yeast two-hybrid assays were performed using four Arabidopsis HD-Zip I TFs. Putative interactions were evaluated by *HIS3* gene activation analysis, and showed that HaHB4.2 was able to interact with AtHB1, AtHB7, AtHB12, and AtHB13 (Fig. 1D).

### Transgenic wheat plants expressing HaHB4.2 exhibit greater yields than controls across contrasting field conditions

**Fig. 2.**
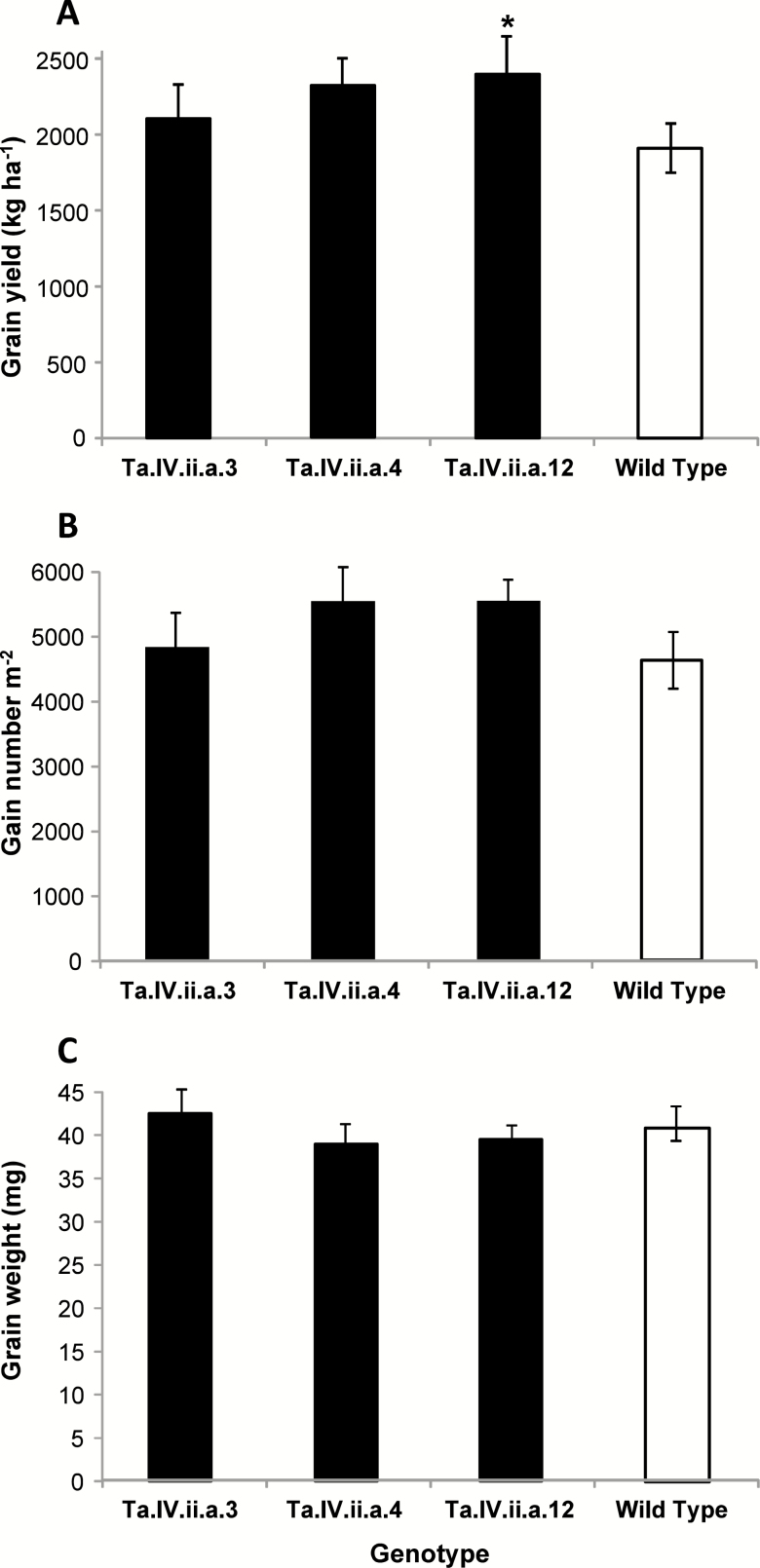
Grain yield and grain yield components of parental cv. Cadenza and three transgenic lines in six field experiments. (A) Grain yield. (B) Grain numbers per m^2^. (C) Individual grain weight. The asterisk in (A) indicate significant difference (*P*<0.05) between Ta.IV.ii.a.12 and the Wild Type. Data correspond to experiments of Group 1 in Supplementary Table S3. Each mean corresponds to 18 items of data (6 experiments×3 replicates). Error bars represent 2×SEM.

From a total of 12 transgenic events obtained, five were homozygous lines with a very high resemblance in phenotypic traits such as occurrence of phenological stages and plant height (Supplementary Table S2). Only the lines identified as Ta.IV.ii.a.3 and Ta.IV.ii.a.11 had a minor delay in the crop cycle and were slightly shorter in plant height as compared with parental cv. Cadenza. Enough seed from three events was obtained to test grain yield performance in six experiments (Group 1), two performed during 2009 (at Villalonga and Monte Buey) and three during 2011 (at Villalonga, Daireaux, and Villa Saboya), including irrigated and non-irrigated conditions in one of them (at Villalonga in 2009). Results from this group, which included three events, indicated significant environment (*P*<0.0001) and genotype (*P*<0.05) effects, but no genotype×environment (*P*>0.05) effect. The line identified as Ta.IV.ii.a.12 was the best performing (Fig. 2A) and the only one that differed significantly from parental cv. Cadenza. Differences in grain yield were supported by similar trends in grain number (Fig. 2B) but not in individual grain weight (Fig. 2C). Based on these results, line Ta.IV.ii.a.12 was registered as IND-ØØ412-7.

**Fig. 3.**
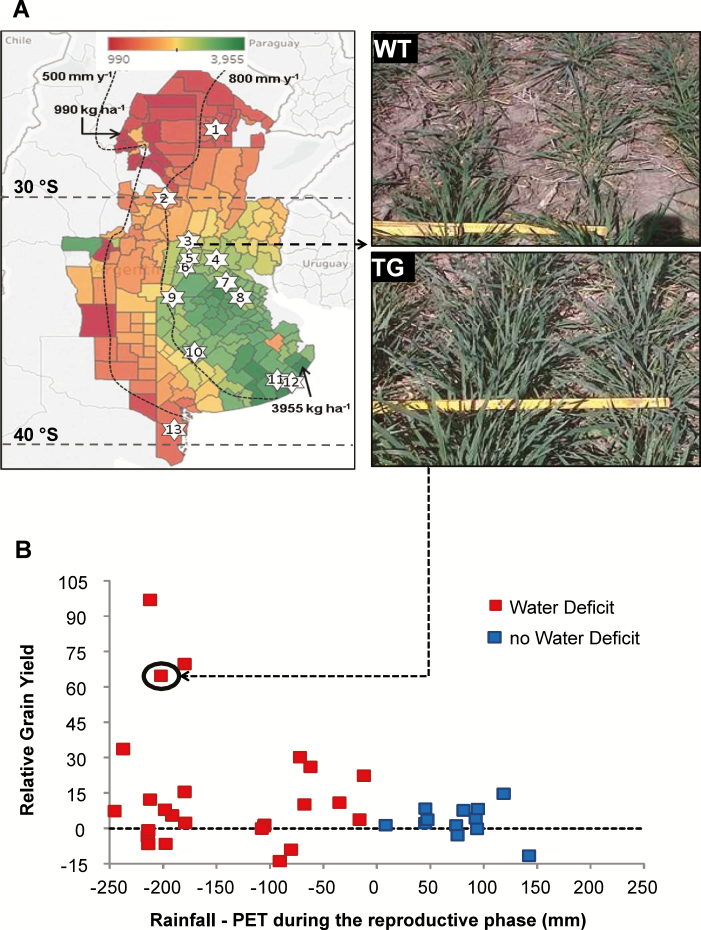
Experimental network and relative grain yield of parental cv. Cadenza and transgenic line IND-OO412-7 across the network. (A) Mean 20-year grain yield (1996/1997–2016/2017) across the wheat production region of Argentina and location of INDEAR evaluation sites. Data are expressed at the department level from a minimum of 990 kg ha^−1^ (dark red) to a maximum of 3955 kg ha^−1^ (dark green), and their distribution matches the estimated drought probability risk for the evaluated region (www.ora.gob.ar). Isohyets of 500 and 800 mm year^−1^ were sketched for reference. Areas in white within the region correspond to departments with less than 10 years of wheat production records (www.agroindustria.gob.ar), and areas in green to the west of the 500 isohyet correspond to irrigated production. The numbered stars inside the map indicate the locations of Indear evaluation sites. 1, Charata; 2, Gutemberg; 3, Landeta; 4, Roldán; 5, Monte Buey; 6, Corral de Bustos; 7, Pergamino; 8, Carmen de Areco; 9, Villa Saboya; 10, Daireaux; 11, Balcarce; 12, Camet; 13, Villalonga. (B) Relative grain yield (RGY=(GY_IND-ØØ412-7−_GY_Cadenza_)/GY_Cadenza_) variation in response to a general water balance computed as the difference between rainfall and potential evapotranspiration (PET) during the reproductive phase (period between anthesis−30 d and maturity). Data correspond to 37 experiments (Group 2 in Supplementary Table S3). Images illustrate WT Cadenza and transgenic (TG) IND-ØØ412-7 plants at tillering corresponding to the encircled value, representative of site 3 on the map (Landeta in 2013).

**Fig. 4.**
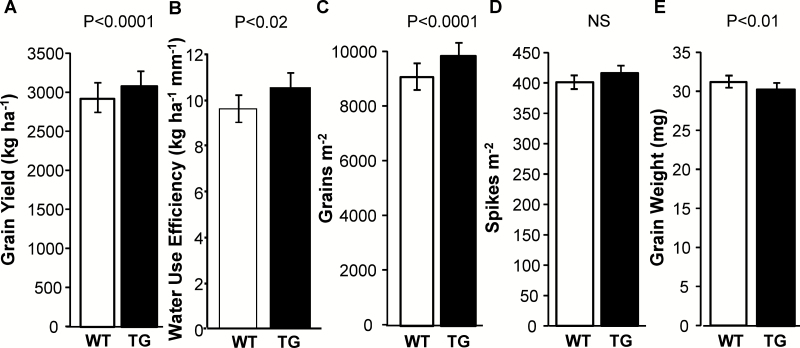
Grain yield and grain yield components of WT cv. Cadenza and TG cv. IND-ØØ412-7. Grain yield (A), water use efficiency (B), grains m^−2^ (C), spikes m^−2^ (D), and individual grain weight (E) of WT and TG crops. Data correspond to the 37 experiments (Group 2 in Supplementary Table S3). Error bars represent 2×SEM.

In the ANOVA performed for grain yield evaluation of cvs Cadenza and IND-ØØ412-7 across 37 environments (Group 2; Supplementary Table S3; Fig. 3A), we detected significant cultivar (*P*<0.001) and environment effects (*P*<0.0001), but no interaction between them (*P*>0.05). Grain yields ranged between (i) 711 and 10 201 kg ha^−1^ for cv. Cadenza grown in experiments performed at Gutemberg (29°43′S, in 2010) and Villalonga (39°52′S, in 2010, experiment ‘b’ in Supplementary Table S3), respectively, and (ii) 1013 and 9515 kg ha^−1^ for cv. IND-ØØ412-7 grown in the experiments conducted at Monte Buey (33°00′S in 2009) and Villalonga (39°52′S in 2010), respectively. Averaged across all experiments, cv. IND-ØØ412-7 outyielded Cadenza by 6% (Fig. 4A). When regressed with respect to the environmental index (average yield across genotypes for each environment; Finlay and Wilkinson, 1963), linear models fitted to cv. IND-ØØ412-7 (*y*=−70.7 + 0.98*x*; *P*<0.001) and Cadenza (*y*=−378 + 1.01*x*; *P*<0.001) did not differ markedly, but the former was above the latter for the whole range of evaluated environments (Supplementary Fig. S1A). Lack of difference between fitted models also held when compared with the mean of local commercial cultivars used as control plots (*y*=216 + 0.96*x*; *P*<0.001), although comparisons must be made with care because different commercial cultivars were used across experiments and some experiments did not include this type of control.

Based on the relative grain yield obtained in each experiment (computed as the difference in grain yield between IND-ØØ412-7 and Cadenza, relative to Cadenza grain yield), mean percentage benefit was larger (16%) in water-deficit environments than in non-stressed environments (3%). The former (represented in red in Fig. 3B) were those where potential evapotranspiration (PET, in mm) exceeded rainfall during the reproductive phase (period between 30 d before anthesis and maturity). Maximum benefit (97%) corresponded to one of these environments (Gutemberg in 2010), with grain yields of 711 kg ha^−1^ for cv. Cadenza and 1399 kg ha^−1^ for cv. IND-ØØ412-7. The opposite trend (rainfall>PET) was verified in 12 of the 37 cases (represented in blue in Fig. 3B), with no significant shift in the predominant grain yield trend between cultivars (IND-ØØ412-7>Cadenza). Only eight out of 37 records registered some degree of grain yield penalization for the transgenic IND-ØØ412-7, which averaged −7%. The largest penalization (−14%) corresponded to the experiment performed at Roldán during 2015 (32°54′S), where cv. Cadenza yielded 2339 kg ha^−1^ and cv. IND-ØØ412-7 yielded 2014 kg ha^−1^. Taking into account the whole dataset, water use efficiency in terms of grain yield adjusted by total rainfall (WUE_GY,R_; Fig. 4B) was 9.4% greater (*P*<0.02) for cv. IND-ØØ412-7 (10.5 kg ha^−1^ mm^−1^) than for cv. Cadenza (9.6 kg ha^−1^ mm^−1^). When the computation was restricted to experiments used to estimate the first stage of potential WUE_GY,R_ (frontier line at low-rainfall environments in Supplementary Fig. S1B), this difference increased to 14.2%. Regression analysis established that most of the grain yield variation was explained by the variation registered in grain numbers (92%; *P*<0.001), and to a lesser extent by the variation registered in individual grain weight (36%; *P*<0.001). For grain numbers, an 8% increase was detected for IND-ØØ412-7 compared with Cadenza (*P*<0.0001, Fig. 4C). No significant difference was detected in spikes m^−2^ (3% increase of IND-ØØ412-7 compared with Cadenza; Fig. 4D), whereas a decrease was detected in individual grain weight (−3% of IND-ØØ412-7 compared with Cadenza; *P*<0.01, Fig. 4E).

### Increased yield of wheat HaHB4 is due to the combination of positive effects registered in most of its ecophysiological determinants

For the four environments explored for physiological evaluation (Group 3), mean grain yield ranged between 4788 and 8269 kg ha^−1^.

The significant difference observed in grain yield between cultivars across 37 experiments held when this reduced set of four experiments was considered (*P*<0.02), with mean values of cv. IND-ØØ412-7 (6723 kg ha^−1^) larger than mean values of cv. Cadenza (6293 kg ha^−1^). No difference was detected between cultivars in harvest index (0.31 for both) and spikes m^−2^ (479 for Cadenza and 477 for IND-ØØ412-7), but cv. IND-ØØ412-7 tended (*P*>0.05) to exceed cv. Cadenza in mean values of (i) total biomass (21 818 versus 20 895 kg ha^−1^), (ii) grain numbers (18 162 versus 17 565 grains m^−2^), and (iii) grain weight (35.9 versus 34.8 mg). Differences in grain yield and trends in grain numbers were significantly (*P*<0.008) supported by measurements of crop growth rate during the critical period, for which cv. IND-ØØ412-7 exceeded cv. Cadenza (22.4 and 15.3 g m^−2^ d^−1^, respectively).

**Fig. 5.**
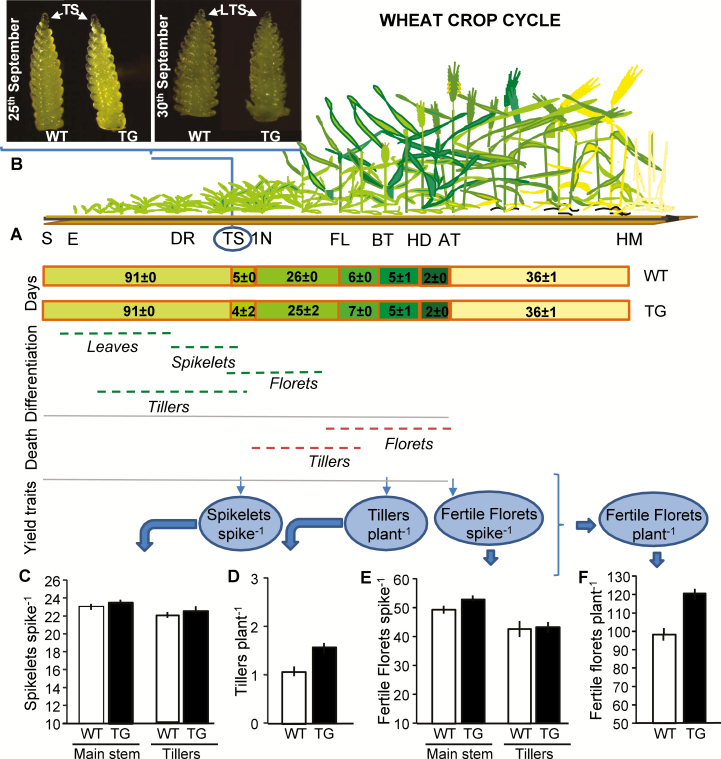
Wheat crop cycle and yield generation. Wheat plants cycle illustration was adapted from Slafer and Rawson (1994). 1N, first node detectable; AT, anthesis; DR, double ridge; E, emergence; FL, flag leaf; HD, heading; HM, harvest maturity; S, sowing; TS, terminal spikelet. (A) Days between external phenological events. (B–F) Apex development at the terminal spikelet (TS) and late terminal spikelet (LTS) stages (B), and determinants of fertile florets plant^−1^ registered on nine plants per genotype (three plants from each replicate) (C–F) for WT Cadenza and transgenic (TG) IND-ØØ412-7 at Pergamino during 2017. Error bars represent 2×SEM.

The presence of HaHB4 had no effect on any of the external phenological events registered during 2017 at Pergamino (Fig. 5A, Group 4), where the cultivars exhibited almost identical anthesis (7 and 6 November for Cadenza and IND-ØØ412-7, respectively) and maturity dates (13 December for both cultivars). Despite no difference being observed in apex development and time to terminal spikelet stage (end of spikelet differentiation, Fig. 5B), cv. IND-ØØ412-7 showed a consistent trend to having main shoot and tiller spikes with larger spikelet number per spike than cv. Cadenza (Fig. 5C). A positive trend was also observed for the number of tillers per plant (Fig. 5D) and fertile florets per spike (Fig. 5E), with enhanced number of fertile florets per plant as final outcome (Fig. 5F). The variation in fertile florets per spike was positively associated with spike weight at anthesis (*R*^2^=45%, *P*=0.086). All these differences in reproductive structures support the improved grain numbers of cv. IND-ØØ412-7 as compared with cv. Cadenza registered across the whole set of experiments (Group 3).

### Transcript levels of *HaHB4* and stress-related genes correlate with yield ecophysiological determinants

**Fig. 6.**
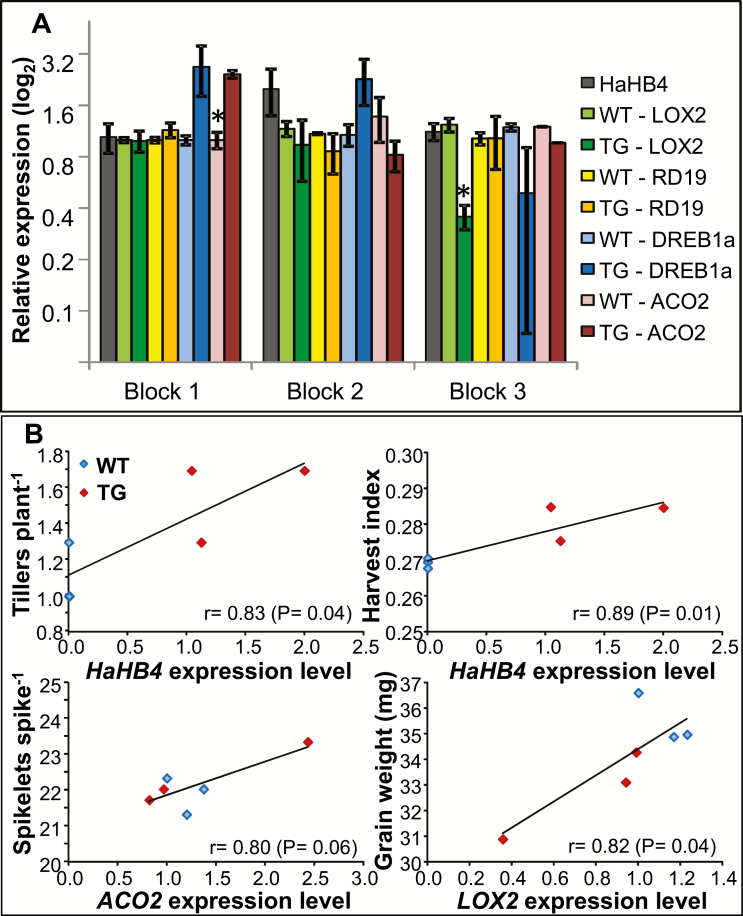
Transcript levels of *HaHB4* and genes related to biotic and abiotic stresses in leaf samples collected in field trials. (A) Relative transcript levels of *HaHB4*, *RD19, DREB1Ba*, and *ACO2* in leaves of 128-day-old plants (i.e. heading of the crops) of wild type Cadenza (WT) and transgenic (TG) IND-ØØ412-7, growing in one experiment (Group 4 in Supplementary Table S3) in different blocks (1, 2, 3). *HaHB4* was related to the lower level, arbitrarily set to a value of 1. All the values were previously related to *ACTIN* level, used as the housekeeping gene. Error bars represent the SEM of three independent biological replicates, each including three technical replicates obtained from a pool of eight plants per plot. Statistical significance was computed by Student’s *t-*test (*P<0.05). (B) Response of ecophysiological traits to the expression level of evaluated genes. Only significant relationships (*P*≤0.06; *n*=6) are shown.

To investigate putative relationships between yield and expression levels of the transgene, leaves of field grown wheat plants were harvested 128 d after sowing (i.e. heading) in Pergamino (in 2017) and total RNA extracted for further analyses by RT-qPCR. Moreover, aiming to understand which molecular mechanisms could be playing a role in yield increase and to test whether plants were stressed or not, several wheat genes were assessed too. We chose two genes (*LOX2* and *ACO2*) with sequence similarity to those previously detected as HaHB4 targets in Arabidopsis and sunflower (Manavella *et al.*, 2006, 2008*b*) and two others (*RD19* and *DREB1a*) considered abiotic stress responsive identified in wheat (Poersch-Bortolon *et al.*, 2016). Leaf samples from each individual block were harvested and pooled to test putative variation between cultivars across blocks. *HaHB4* transcript levels varied from 1 to 2 (approximately 100%) across blocks, whereas *LOX2*, *RD19*, *DREB1a*, and *ACO2* also differed, maintaining a complex relationship with *HaHB4* expression (Fig. 6A). Correlation analysis was performed between the expression level of mentioned genes and all evaluated ecophysiological determinants of grain yield. Significant relationships (*P*≤0.06) were established only with *HaHB4* and putative HaHB4-target genes (Fig. 6B), whereas no relationship was detected for the abiotic stress responsive genes *RD19* and *DREB1a*. The most significant responses to *HaHB4* expression were those detected for harvest index (*P*=0.01), crop growth rate during the critical period (*P*=0.01) and number of tillers plant^−1^ (*P*=0.04). Trends detected for *ACO2* and *LOX2* expression were forced by a large increase (*ACO2*) or decrease (*LOX2*) in only one TG block.

## Discussion

HaHB4 is a TF belonging to the HD-Zip I family, and it was previously shown that encoded by a transgene in Arabidopsis, it was able to confer drought tolerance without yield penalty when its expression was driven by inducible or constitutive promoters (Dezar *et al.*, 2005; Cabello *et al.*, 2007). Such drought tolerance is due, at least in part, to the inhibition of ethylene perception delaying senescence (Manavella *et al.*, 2006). However, these beneficial traits were observed in the model plant, which is a cruciferous dicot. Whether the displayed molecular mechanisms are conserved between species, in particular evolutionarily distant plant species, is still an open question. This is because even though the differential transcriptome presented by *HaHB4*-transgenic plants was deeply analysed and target genes identified (Manavella *et al.*, 2006, 2008a, b; Moreno Piovano *et al.*, 2017), the way this TF is affecting such transcriptome is yet unknown.

Several hypotheses are possible; among them we can state that the TF is activating/repressing specific targets that modulate the proteome and metabolome or that the TF interacts with wheat HD-Zip I TFs, which prevents the latter functioning correctly. Supporting the first hypothesis, HaHB4 was able to transactivate in yeast cells suggesting that it may be affecting transcript levels of wheat target genes (Fig. 1). This activity was slightly affected by mutations in its C-terminus; however, the differences between HaHB4 variants were not large and none of the constructs lacks this capability. Supporting the second hypothesis, HaHB4 was able to interact with different HD-Zip I TFs from Arabidopsis suggesting that this TF may be capturing by dimerization its wheat homologues (Fig. 1). Further experimental work must be carried out to elucidate whether these two mechanisms occur in parallel or only one of them is occurring.

Crop yield is a complex trait that depends on several parameters, both genetic and environmental, that affect crop potential capability. Transgenic wheat expressing the sunflower *HaHB4* exhibited a drought-tolerant phenotype in different environments, which triggered a yield increase in such conditions (Figs 2, 3). It is important to note that *HaHB4*, although a transgene in wheat, is a gene coming from sunflower, a species usually cropped in drought-prone areas and used to feed humans as well as animals. Safety assessments requested by worldwide agencies were performed and showed that the nutritional value of this wheat is equivalent to that of its WT Cadenza (Ayala *et al.*, 2019). Moreover, all the metabolites whose levels were changed in the presence of HaHB4 are natural components of wheat and normally varied with environmental or hormonal changes that plants usually experience. Microarray analyses performed with non-transgenic wheat plants in different environments or along day and night cycles indicated strong changes in the corresponding transcriptomes and metabolomes (Abdeen *et al.*, 2010).

Contrary to recent evidence of null or negative effects on production traits due to the overexpression of class I homeodomain TF TaHDZipI-5 in wheat plants grown under irrigated and mild water deficit in controlled environments (Yang *et al.*, 2018), we clearly demonstrated the benefit of the expression of HaHB4 TF in wheat crops in a large net of 37 field trials, developed on representative agricultural soils and using common agronomic practices (Fig. 3). Moreover, the mentioned benefit allowed the transformed variety to achieve grain yields similar to different commercial controls included in experiments, which were comparatively modern local varieties expected to have improved adaptation as well as yield potential (Cadenza was released to the UK market in 1995, and is not among the best adapted cultivars to Argentine wheat environments mainly due to its long cycle). Studies on transgenic events aimed at drought-prone environments usually fail to achieve in field-grown plots the benefits observed when plants are grown in water-deficit conditions in controlled environments. Failure comes, mostly, from the poor understanding of the physiology of crop grain yield determination and lack of a correct phenotyping for breeding purposes (Passioura, 2012), which demands a shift of focus from survival traits usually surveyed in individual plants to production traits measured on a per unit land basis (i.e. plants in community). Scaling up from plant to crop is typically linked to many trade-offs that customarily cancel the expected benefits in final grain yield (Passioura, 2010). For a transgenic event to be of value at the crop level, its effects must translate into increased biomass production and/or biomass partitioning to grains (Passioura, 2012) with no change in crop phenology, provided anthesis date has been already optimized (Richards, 2006). Our robust finding of enhanced grain yield linked to the expression of *HaHB4* in field conditions was supported by the increase in two important traits: water use efficiency and grain numbers (Fig. 4). The former, based on grain yield production per unit of total rainfall during the cycle (French and Schultz, 1984; Sinclair *et al.*, 1984), was clearly augmented for the transgenic cultivar, particularly in low-rainfall environments. The latter was the main determinant of wheat grain yield, and its increase was not cancelled by trade-off effects in individual grain weight (Griffiths *et al.*, 2015; Kovalchuk *et al.*, 2016). Such responses, which predominated across 28 experiments of the global net, are the expected result of improved biomass production and/or biomass partitioning to reproductive structures during reproductive stages (Unkovich *et al.*, 2010), chiefly the critical period for grain set that spans the 30 d before anthesis (Kirby and Appleyard, 1987). Improved crop growth during this period translates into improved spike biomass, floret survival, and fertile floret number at anthesis (González *et al.*, 2011). In this context, significant differences (e.g. in crop growth rate during the critical period) and positive trends (e.g. in total biomass at maturity, spike weight at anthesis, fertile floret number per spike) observed in related secondary traits surveyed in a limited set of experiments support the clear increase registered in grain numbers and grain yield of the cultivar expressing the HaHB4 TF (Fig. 5).

The expression of *HaHB4* in wheat grown in the field was evaluated together with the expression of four other genes (*ACO2*, *LOX2*, *RD19*, and *DREB1a*), which resulted in different levels in each individual plot. This variable response within each plot was probably due to unevaluated conditions of soil and air humidity. The most noticeable aspect was the significant relationships between important determinants of grain yield (e.g. harvest index) and expression levels of *HaHB4* and HaHB4-target genes (*ACO2* and *LOX2*). *LOX2* encodes an enzyme participating in jasmonic acid biosynthesis, which was shown to be induced in *HaHB4* transgenic Arabidopsis and maize plants (Manavella *et al.*, 2008*b*). However, transcript levels of these genes evaluated in this work showed a slight tendency to be reduced compared with the WT (Fig. 6). This apparent discrepancy could be explained by the fact that they can be naturally induced as a protective response in field conditions. *RD19* (encoding a cysteine protease; Koizumi *et al.*, 1993) and *DREB1a* (a TF) are genes reported to be strongly regulated by water deficit conditions and involved in this and other abiotic stress responses (Shinozaki and Yamaguchi-Shinozaki, 2000). Transcripts of *RD19* were almost unchanged in high yielding blocks and slightly reduced in transgenic plants of the low-yielding ones, whereas *DREB1a* followed the opposite trend. These results suggest that action of HaHB4 is not dependent on the response triggered either by *RD19* or by *DREB1a*.

Altogether, our results indicate that transgenic *HaHB4* wheat plants can be used to improve yield in this species relevant for world food security, especially in marginal regions characterized by frequent drought events. Current breeding efforts are aimed at the transformation with *HaHB4* of modern elite cultivars with improved adaptation to the Argentine market. This transgenic wheat has been approved for biosafety (CONABIA, Argentinean Ministry of Agriculture) and food safety (SENASA, Argentinean Ministry of Agriculture). However, commercial release is still pending and depends on a government political decision that has not been taken yet.

## Supplementary data

Supplementary data are available at *JXB* online.

Fig. S1. Grain yield response across environments.

Table S1. Oligonucleotides used for cloning and RT-qPCR.

Table S2. Phenology and plant height of parental cv. Cadenza and five transgenic events.

Table S3. Environmental description of experiments.

Supplemental Tables S1-S3 and Figures S1
