# Supplementary material for: Field-grown transgenic wheat expressing the sunflower gene HaHB4 significantly outyields the wild type
Source: J Exp Bot. 2019 Feb 6;70(5):1669–81. doi: 10.1093/jxb/erz037 (PMC6411379; doi:10.1093/jxb/erz037)
Supplement: Supplemental Tables S1-S3 and Figures S1 [file erz037_suppl_supplementary_tables_s1_s3_figure_s1.pdf]

Wheat transgenic plants expressing the sunflower gene *HaHB4* significantly outyielded their controls in field trials

Fernanda Gabriela González[ac#], Matías Capella[b#], Karina Fabiana Ribichich[b], Facundo Curín[c], Jorge Ignacio Giacomelli[b], Francisco Ayala[d], Gerónimo Watson[d], María Elena Otegui[e*], Raquel Lía Chan [b*]

Table S1. Oligonucleotides used for cloning and gene expression analysis.

Name	ID	Sequence 5'-→3'	Use for
H4m-F	AF339748 and AF339749	ATGTCTCTTCAACAAGTAACAACCACCAGG	Deletion of the segment PTTE in the NH_2. Provides ATG.
H4m-R	AF339748 and AF339749	TTAGAACTCCCACCACTTTTGAAGGTCTGGC	Change a Pro by a Leu in the COO^-. Provides stop codon
H4m-F1	AF339748 and AF339749	CGGTTTTGCGTACCTTGAAGAAGGAAACAGTTTG	Change a Phe by a Leu (chimerical construct)
H4m-R 1	AF339748 and AF339749	GTTTCCTTCTTCAAGGTACGCAAAACCGTCGC	Change a Phe by a Leu in the complementary strand (chimerical construct)
Transf1	AF339748 and AF339749	GCGGGATCCACCATGTCTCTTCAACAAGTA	Provides *BamH*1 site, translational enhancer ACC and ATG
Transf2	AF339748 and AF339749	GCCGAGCTCTTAGAACTCCCACCACTTTTG	Provides *Sac*I site and stop codon
HaHB4qF	AF339748 and AF339749	gggCTTCATCCTCgTCAAgTggC	qPCR of HaHB4
HaHB4qR	AF339748 and AF339749	ACgCAAgCgTCTCgTAgTTATg	qPCR of HaHB4
TaLOX2qF	scfld336632_5BS	GGCATCCCCAACAGCATCTCC	qPCR of LOX2
TaLOX2qR	scfld361901_5DS	GATGTCCCATTCCAATTATACCC	qPCR of LOX2

TaRD19qF	scfld374347_5DL<br>scfld346111_5BL<br>scfld321097_5AL	GTCCCTCCAGTCGAAGTCCT	qPCR of RD19
TaRD19qR		TCACCAAGTTCTCCGACCTC	qPCR of RD19
TaDREB1aqF	scfld322800_5AL	GTAGGGGATTCGCCTTCTTC	qPCR of DREB1a
TaDREB1aqR		GGAACGGGAACTTGACAAAC	qPCR of DREB1a
TaACO2qF	scfld432067_6BL	TGATCCTGCTGTTTCAGGAC	qPCR of ACO2
TaACO2qR		GTAGAAGGAGGCGATGGACA	qPCR of ACO2
TaACTqF	scfld453873_6DL<br>scfld348405_5BL	GGTAACATTGTGCTCAGTGGTGG	qPCR of ACT
TaACTqR		CACCACCTTGATCTTCATGCTGC	qPCR of ACT

The complete accession number (ID) begins with the sentence **gnl|Ta_Cdnza|whe_Ta_ABD_Cadenza-EIv1_2017_** followed by the scaffold (scfld) number. Accession numbers are from GrainGenes database (The *T. aestivum* cv. Cadenza (Earlham Inst. Scaffolds, 2017) wheat collection at https://wheat.pw.usda.gov/GG3/). Oligonucleotides for *RD19* and *DREB1a* qPCR were described in Poersch-Bortolon et al. (2016).

References:

-GrainGene database. The *T. aestivum* cv. Cadenza (Earlham Inst. Scaffolds, 2017) wheat collection, https://wheat.pw.usda.gov/GG3/

-Poersch-Bortolon, LB; Pereira, JF; Nhani Junior, A; Gonzáles, HHS; Torres, GAM; Consoli, L; Arenhart, RA; Bodanese-Zanettini, MH & Margis-Pinheiro, M. (2016). Gene expression analysis reveals important pathways for drought response in leaves and roots of a wheat cultivar adapted to rainfed cropping in the Cerrado biome. Genetics and Molecular Biology, 39(4), 629-645. https://dx.doi.org/10.1590/1678-4685-gmb-2015-0327

Wheat transgenic plants expressing the sunflower gene *HaHB4* significantly outyielded their controls in field trials

Fernanda Gabriela González[ac#], Matías Capella[b#], Karina Fabiana Ribichich[b], Facundo Curín[c], Jorge Ignacio Giacomelli[b], Francisco Ayala[d], Gerónimo Watson[d], María Elena Otegui[e*], Raquel Lía Chan [b*]

Supplementary Table 2.

Mean and standard error (SE) of phenological events (based on Zadoks, 1974) and plant height of wild type cv. Cadenza and five transgenic events. The number of evaluated plants is indicated in parenthesis.

PHENOLOGY		Days after sowing																	
**Cultivar**		**12**	**20**	**27**	**34**	**42**	**49**	**57**	**63**	**70**	**77**	**84**	**91**	**98**	**105**	**111**	**119**	**126**	**131**
Cadenza WT (n= 2)	Mean	1.1	1.3	1.4	2.1	2.2	2.4	2.5	2.6	2.6	3.1	3.7	5.5	5.9	7.3	8.5	8.7	8.9	9.2
	SE	0.00	0.00	0.00	0.00	0.00	0.00	0.00	0.00	0.00	0.00	0.00	0.00	0.00	0.00	0.00	0.00	0.00	0.00
Ta.IV.ii.a.3 (n= 10)	Mean	1.1	1.3	1.4	2.13	2.23	2.4	2.5	2.6	2.6	3.12	3.7	5.3	5.86	7.16	8.36	8.6	8.86	9.17
	SE	0.00	0.00	0.00	0.05	0.05	0.00	0.00	0.00	0.00	0.04	0.00	0.42	0.13	0.44	0.38	0.32	0.13	0.09
Ta.IV.ii.a.4 (n= 10)	Mean	1.1	1.3	1.4	2.1	2.2	2.4	2.5	2.6	2.6	3.1	3.7	5.5	5.9	7.3	8.5	8.7	8.9	9.2
	SE	0.00	0.00	0.00	0.00	0.00	0.00	0.00	0.00	0.00	0.00	0.00	0.00	0.00	0.00	0.00	0.00	0.00	0.00
Ta.IV.ii.a.6 (n= 15)	Mean	1.1	1.3	1.4	2.1	2.21	2.4	2.5	2.6	2.6	3.11	3.7	5.5	5.9	7.3	8.5	8.7	8.9	9.2
	SE	0.00	0.00	0.00	0.00	0.03	0.00	0.00	0.00	0.00	0.03	0.00	0.00	0.00	0.00	0.00	0.00	0.00	0.00
Ta.IV.ii.a.11.2 (n= 11)	Mean	1.1	1.3	1.4	2.12	2.2	2.4	2.5	2.6	2.6	3.1	3.7	5.39	5.9	7.3	8.48	8.68	8.88	9.17
	SE	0.00	0.00	0.00	0.04	0.00	0.00	0.00	0.00	0.00	0.00	0.00	0.36	0.00	0.00	0.06	0.06	0.06	0.09
Ta.IV.ii.a.12 (n= 11)	Mean	1.1	1.3	1.4	2.1	2.2	2.4	2.5	2.6	2.6	3.14	3.7	5.5	5.9	7.3	8.5	8.7	8.9	9.2
	SE	0.00	0.00	0.00	0.00	0.00	0.00	0.00	0.00	0.00	0.05	0.00	0.00	0.00	0.00	0.00	0.00	0.00	0.00

PLANT HEIGHT (cm)		Days after sowing																	
**Cultivar**		**12**	**20**	**27**	**34**	**42**	**49**	**57**	**63**	**70**	**77**	**84**	**91**	**98**	**105**	**111**	**119**	**126**	**131**
Cadenza WT (n= 2)	Mean	5	9.5	13	15	17	23	26	30	40	45	65	77	85	90	90	90	90	90
	SE	0.00	2.12	1.41	1.41	1.41	0.00	0.00	0.00	0.00	0.00	0.00	0.00	0.00	0.00	0.00	0.00	0.00	0.00
Ta.IV.ii.a.3 (n= 10)	Mean	4.6	9.8	12.7	14.5	16.8	23.5	26.3	29.7	38.4	43.9	59.9	70.2	77.8	82.2	82.2	82.2	82.2	82.2
	SE	0.84	2.39	1.70	1.84	2.15	1.84	1.77	1.77	3.86	2.85	10.88	14.71	16.37	17.90	17.90	17.90	17.90	17.90
Ta.IV.ii.a.4 (n= 10)	Mean	5	8.9	12.5	14.6	16.6	23	26	30	40	45	65	77	85	90	90	90	90	90
	SE	0.00	1.52	0.85	0.97	0.97	0.00	0.00	0.00	0.00	0.00	0.00	0.00	0.00	0.00	0.00	0.00	0.00	0.00
Ta.IV.ii.a.6 (n= 15)	Mean	5	8.13	12.1	14.1	16.1	23	26	30	40	45	65	77	85	90	90	90	90	90
	SE	0.00	0.52	0.52	0.52	0.52	0.00	0.00	0.00	0.00	0.00	0.00	0.00	0.00	0.00	0.00	0.00	0.00	0.00
Ta.IV.ii.a.11.2 (n= 11)	Mean	4.91	8.27	12	13.9	16.3	23	25.8	30	40	45	65	74.9	83.6	89.1	89.1	89.1	89.1	89.1
	SE	0.30	0.65	1.18	0.94	0.90	0.00	0.40	0.00	0.00	0.00	0.00	6.93	4.52	3.02	3.02	3.02	3.02	3.02
Ta.IV.ii.a.12 (n= 11)	Mean	5	10	14	16	18	23	26	30	40	45	65	77	85	90	90	90	90	90
	SE	0.00	0.00	0.00	0.00	0.00	0.00	0.00	0.00	0.00	0.00	0.00	0.00	0.00	0.00	0.00	0.00	0.00	0.00

Wheat transgenic plants expressing the sunflower gene *HaHB4* significantly outyielded their controls in field trials

Fernanda Gabriela González[ac#], Matías Capella[b#], Karina Fabiana Ribichich[b], Facundo Curín[c], Jorge Ignacio Giacomelli[b], Francisco Ayala[d], Gerónimo Watson[d], María Elena Otegui[e*], Raquel Lía Chan[b*]

Table 1. Management and environmental characteristics of Bioceres wheat evaluation network. Underlined values correspond to maximum and minimum records of each variable across all experiments. Bolded data correspond to cases with Tmax > 30 °C (indicative of heat stress) and Cumulative PET > Cumulative Rainfall (indicative of increased probability of water deficit). Experiments were organized in four groups depending upon the aim of the study. Group 1: experiments 7, 24, 27, 33, 34, 37. Group 2: all experiments. Group 3: experiments 6, 13, 20, 21. Group 4: experiment 21.

Supplementary Figure 1.

Grain yield (GY) response to (A) the environmental index (EI), and (B) total rainfall (R) during the cycle. The environmental index was computed as the mean grain yield of all cultivars evaluated in a given environment, and included cv. IND-ØØ412-7 as well as parental cv. Cadenza and a variable number (between 0 and 6) of commercial controls tested in each environment. Dotted lines in (A) represent the linear models fitted to each group. Slopes of the dashed line in (B) represent the potential wáter use efficiency (French and Schultz, 1984) based on full symbols (n= 9), which were also identified in (A). For all regressions, P≤ 0.001.

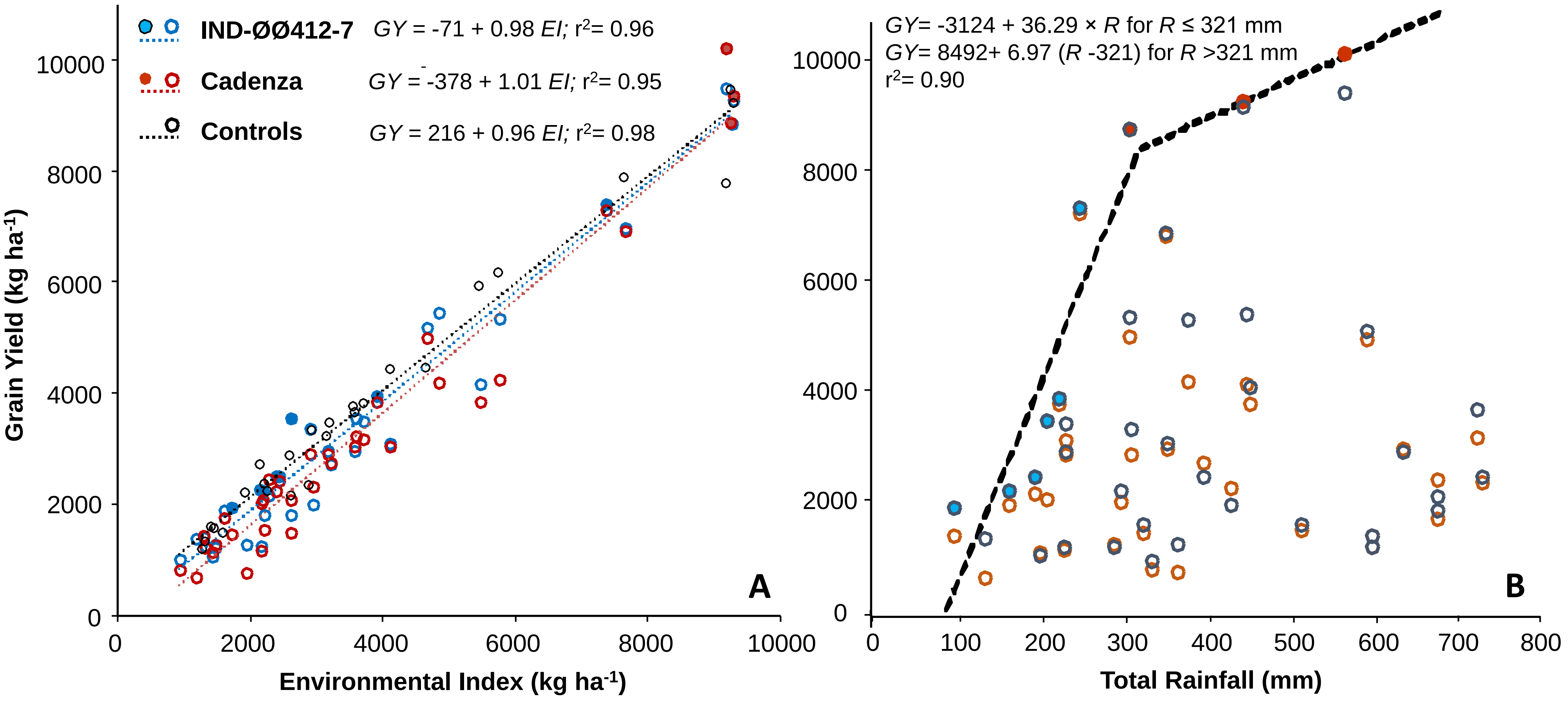

Experiments	South Latitud	West Longitude	Year	Sowing Date	Anthesis Date	Harvest Date	Critical Period						Grain-Filling Period					
							Mean Tmax (°C)	Tmean (°C)	Cumulative PAR (MJ m^{-2})	Photothermal quotient (MJ °Cday^{-1})	Cumulative Rainfall (mm)	Cumulative PET (mm)	Mean Tmax (°C)	Tmean (°C)	Cumulative PAR (MJ m^{-2})	Photothermal quotient (MJ °Cday^{-1})	Cumulative Rainfall (mm)	Cumulative PET (mm)
1-Charata	27° 14'	61° 11'	2010	1-Jul	11-Oct	7-Dec	27.7	20.8	274.1	0.40	17.6	**120.6**	**30.4**	23.0	296.6	0.45	45.3	**154.5**
2-Gutemberg	29° 43'	63° 30'	2010	24-Jun	15-Oct	14-Dec	29.5	21.0	269.1	0.43	47.6	**126.3**	**32.9**	24.0	349.7	0.50	43.2	**176.7**
3-Landeta	32° 00'	62° 03'	2013	10-Jun	22-Oct	11-Dec	27.1	18.6	292.1	0.56	27.7	**159.9**	29.5	21.4	333.5	0.52	122.5	192.3
4-Roldán	32° 54'	60° 59'	2014	21-Jul	07-Nov	n/a	29.9	21.3	305.1	0.48	10.7	**145.5**	**32.7**	23.4	352.4	0.51	97.4	177.0
5-Roldán	32° 54'	60° 59'	2015	9-Jun	22-Oct	21-Dec	20.8	15.0	218.7	0.50	30.0	**105.9**	24.1	19.0	230.0	0.41	111.9	126.6
6-Roldán	32° 54'	60° 59'	2016	27-May	17-Oct	7-Dec	24.2	17.2	248.9	0.47	119.0	105.9	27.4	20.4	293.9	0.49	61.0	**136.2**
7-Monte Buey	33° 00'	62° 25'	2009	28-Jun	31-Oct	22-Dec	27.2	19.2	289.8	0.52	78.0	120.6	28.3	22.1	280.4	0.43	159.0	128.4
8-Monte Buey	33° 00'	62° 27'	2012a	31-May	14-Oct	26-Dec	22.8	16.5	225.9	0.50	153.5	85.2	26.7	20.7	287.1	0.47	129.2	123.0
9-Monte Buey	33° 00'	62° 27'	2012b	19-Jun	25-Oct	26-Dec	23.3	17.6	234.9	0.47	205.2	92.7	28.8	22.2	322.7	0.50	122.5	141.0
10-Monte Buey	33° 00'	62° 27'	2013	14-Jun	27-Oct	12-Dec	26.2	18.7	275.4	0.51	27.5	**128.1**	28.6	22.1	287.6	0.43	296.0	149.9
11-Monte Buey	33° 00'	62° 27'	2014	14-Jul	10-Nov	n/a	28.4	20.3	302.9	0.50	64.1	**141.3**	**31.4**	22.6	367.2	0.55	60.2	**180.6**
12-Monte Buey	33° 00'	62° 27'	2015	27-May	04-Oct	16-Jan	21.6	15.0	207.0	0.49	49.0	79.5	23.1	17.4	236.7	0.46	57.9	107.7
13-Monte Buey	33° 00'	62° 27'	2016	26-May	12-Oct	6-Dec	24.4	17.0	250.7	0.50	39.0	**98.7**	25.4	19.1	234.5	0.41	61.0	106.8
14-Corral de Bustos	33° 16'	62° 07'	2010	1-Jun	23-Oct	30-Dec	22.2	15.8	237.2	0.51	88.3	83.4	28.3	19.8	320.9	0.55	23.0	**132.9**
15-Corral de Bustos	33° 16'	62° 07'	2011	30-May	16-Oct	14-Dec	27.5	20.5	310.1	0.51	26.0	**131.7**	**30.4**	23.1	338.0	0.50	80.3	153.9
16-Corral de Bustos	33° 16'	62° 08'	2012	30-May	13-Oct	12-Dec	22.8	16.4	224.1	0.50	115.5	84.0	26.9	20.9	285.3	0.46	167.2	123.0
17-Corral de Bustos	33° 16'	62° 07'	2013	17-Jun	27-Oct	17-Dec	26.2	18.7	275.9	0.51	27.5	**128.1**	28.6	22.1	287.6	0.43	296.0	150.0
18-Corral de Bustos	33° 16'	62° 07'	2014	15-Jul	11-Nov	n/a	28.5	20.5	303.3	0.50	64.1	**142.2**	**31.1**	22.6	360.5	0.54	64.0	**177.3**
19-Pergamino	33° 51'	60° 32'	2015	28-May	16-Oct	21-Dec	21.5	14.5	231.3	0.54	66.2	89.7	26.9	19.3	271.8	0.48	129.6	122.1
20-Pergamino	33° 51'	60° 32'	2016	20-May	04-Nov	7-Dec	24.0	17.3	272.3	0.97	118.0	115.5	**31.7**	23.1	347.0	0.86	61.0	**171.0**
21-Pergamino	33° 51'	60° 32'	2017	26-Jun	05-Nov	13-Dec	23.1	17.1	293.0	0.58	56.2	**116.4**	27.4	17.9	460.2	0.70	38.9	**223.9**
22-Carmen de Areco	34° 19'	59° 50'	2012a	14-Jun	30-Oct	4-Jan	24.0	17.8	242.6	0.46	298.6	104.4	**30.1**	22.3	334.8	0.51	111.1	162.6
23-Carmen de Areco	34° 19'	59° 50'	2012b	6-Jul	09-Nov	4-Jan	27.1	20.1	282.2	0.47	232.9	130.8	29.9	22.4	341.6	0.51	144.4	165.3
24-Villa Saboya	34° 29'	62° 42'	2011	17-Jun	25-Oct	19-Dec	27.1	19.4	244.8	0.42	150.2	111.3	**32.9**	24.4	334.8	0.47	95.6	169.5
25-Villa Saboya	34° 29'	62° 42'	2012	7-Jun	24-Oct	22-Dec	24.2	17.3	240.3	0.48	224.9	101.7	**30.3**	22.8	311.0	0.46	123.0	153.6
26-Villa Saboya	34° 29'	62° 42'	2013	26-Jun	07-Nov	12-Dec	28.2	20.1	273.6	0.46	220.3	125.1	**30.8**	22.3	350.6	0.52	85.1	**171.9**
27-Daireaux	36° 37'	62° 05'	2011	23-Jun	13-Nov	29-Dec	24.9	17.6	297.5	0.58	320.0	128.1	29.2	22.0	362.3	0.55	77.0	**174.3**
28-Daireaux	36° 37'	62° 04'	2012	12-Jun	01-Nov	28-Dec	21.9	17.3	240.8	0.47	244.0	102.6	26.7	21.4	313.7	0.50	125.0	147.6
29-Daireaux	36° 37'	62° 05'	2013	27-Jun	13-Nov	19-Dec	24.9	18.4	261.5	0.48	154.0	114.6	29.0	21.4	347.4	0.54	58.0	**165.0**
30-Balcarce	37° 53'	58° 19'	2012	25-Jul	05-Dec	7-Jan	25.6	18.0	339.3	0.66	108.8	145.8	26.8	19.3	340.2	0.60	236.6	152.1
31-Balcarce	37° 53'	58° 19'	2013	26-Jul	12-Dec	13-Jan	26.4	18.1	360.0	0.68	123.7	156.6	**32.3**	22.9	376.2	0.56	19.3	**184.5**
32-Camet	37° 54'	57° 40'	2011	6-Jul	02-Dec	16-Jan	23.2	18.3	214.7	0.41	87.0	93.3	24.1	19.9	208.4	0.35	30.0	**95.7**
33-Villalonga	39° 52'	62° 40'	2009a	13-Jul	27-Nov	4-Jan	24.7	17.4	285.8	0.57	37.1	**129.9**	27.5	20.3	321.8	0.54	66.0	**153.0**
34-Villalonga	39° 52'	62° 40'	2009b	13-Jul	27-Nov	4-Jan	24.7	17.4	285.8	0.57	37.1	**129.9**	27.5	20.3	321.8	0.54	66.0	**153.0**
35-Villalonga	39° 52'	62° 40'	2010a	24-Jun	24-Nov	10-Jan	23.7	17.2	270.9	0.54	67.7	**122.7**	27.9	19.9	345.2	0.59	19.0	**177.9**
36-Villalonga	39° 52'	62° 40'	2010b	24-Jun	24-Nov	10-Jan	23.7	17.2	270.9	0.54	67.7	**122.7**	27.9	19.9	345.2	0.59	19.0	**177.9**
37-Villalonga	39° 52'	62° 40'	2011	12-Jul	22-Nov	23-Dec	26.9	19.2	305.1	0.55	20.5	**139.8**	27.9	20.4	336.2	0.56	46.0	**163.8**
